# A New Genetic Linkage Map of the Zygomycete Fungus *Phycomyces blakesleeanus*

**DOI:** 10.1371/journal.pone.0058931

**Published:** 2013-03-14

**Authors:** Suman Chaudhary, Silvia Polaino, Viplendra P. S. Shakya, Alexander Idnurm

**Affiliations:** Division of Cell Biology and Biophysics, School of Biological Sciences, University of Missouri-Kansas City, Kansas City, United States of America; University of Nebraska, United States of America

## Abstract

*Phycomyces blakesleeanus* is a member of the subphylum Mucoromycotina. A genetic map was constructed from 121 progeny of a cross between two wild type isolates of *P. blakesleeanus* with 134 markers. The markers were mostly PCR-RFLPs. Markers were located on 46 scaffolds of the genome sequence, covering more than 97% of the genome. Analysis of the alleles in the progeny revealed nine or 12 linkage groups, depending on the log of the odds (LOD) score, across 1583.4 cM at LOD 5. The linkage groups were overlaid on previous mapping data from crosses between mutants, aided by new identification of the mutations in primary metabolism mutant strains. The molecular marker map, the phenotype map and the genome sequence are overall congruent, with some exceptions. The new genetic map provides a genome-wide estimate for recombination, with the average of 33.2 kb per cM. This frequency is one piece of evidence for meiosis during zygospore development in Mucoromycotina species. At the same time as meiosis, transmission of non-recombinant chromosomes is also evident in the mating process in *Phycomyces*. The new map provides scaffold ordering for the genome sequence and a platform upon which to identify the genes in mutants that are affected in traits of interest, such as carotene biosynthesis, phototropism or gravitropism, using positional cloning.

## Introduction

The fungus *Phycomyces blakesleeanus* is a member of the order Mucorales in the subphylum Mucoromycotina. By current estimates the basal lineages in the fungal kingdom, like the Mucoromycotina, represent less than 5% of all fungal species and yet hold the majority of the evolutionary history in this group of eukaryotes [1], [2], [3]. Estimating overall diversity at the DNA level or dating divergence is a challenge [4], although each lineage diverged prior to the split between the Ascomycota and Basidiomycota. Nevertheless, the early branches in the fungal kingdom receive relatively little research attention, despite our potential ability to understand the evolution of the fungi through their study.

This paucity in research effort is surprising in the context of the long research history associated with some Mucoromycotina species, especially on the mating properties of fungi. The first report of sex in fungi comes from the Mucorales [5], [6], [7]. The two major mating strategies in fungi, i.e. homothallism or heterothallism, were defined in the Mucorales [8]. Diffusible pheromones in fungi were first identified from *Mucor mucedo* [9]. However, the genes required for mating in the Mucorales are poorly understood, and completing the sexual cycle under laboratory conditions is often a challenge. In addition to these obstactles, many species are difficult to manipulate via the introduction of foreign DNA. For example, the current absence of a stable transformation system in *Phycomyces* prevents the use of techniques such as cloning by complementation or gene function testing via disruption or RNAi-based silencing [10].

*Phycomyces* reproduces as a mycelium and forms spores either asexually or sexually. The asexual cycle includes the production of spores borne on sporangiophores that may rise 10–15 cm from the surface of the fungal colony. These long filaments are sensitive to many environmental conditions, including light that is a signal to trigger a phototropic response [11], [12]. The sexual cycle leads to the production of zygospores and then progeny, a cycle defined by the 1920s as follows [13]. Two hyphae from each mating type encounter each other, the tips of which undergo a septation event to produce the equivalent of gametes. The two cells fuse to form the immature zygospore. The developing zygospore initially incorporates thousands of nuclei from the two original parents. During the course of zygospore maturation and dormancy, of two months to more than a year, most nuclei degrade. It is hypothesized that two nuclei, one from each parent, survive, fuse and undergo meiosis. Mitotic divisions then amplify the four meiotic products that form in a sporangium structure that develops out from the zygospore. However, analysis of genetic markers in progeny from crosses shows that the situation outlined above does not occur for all zygospores [14], [15], [16], [17]. Furthermore, sex heterokaryons also arise, which are easily detected due to the production of the curled pseudophore structures that resemble gametangia [18]. Indeed, heterokaryon formation is used as a tool since they can be applied in complementation tests involving both nutritional and phototropic markers [19]. Because of the non-Mendelian ratios of genetic markers in progeny from individual zygospores, the presence of meiosis in *Phycomyces* has been challenged, most recently in reference [17].

Strains of *Phycomyces* with mutant phenotypes were isolated by chemical mutagenesis. Whatever the mechanism(s) of recombination in the zygospore, individual genes could then be defined into complementation groups and their relative placement on chromosomes established through genetic linkage analysis. Over a two decade period this process led to the measurement of linkage distances based on segregation between these markers [20], [21], [22], [23], [24], [25], [26], including centromeres that were mapped with the equivalent of tetrad analysis [27], [28]. The first genetic map was reported in 1987 [28], and the updated map was published in 1996 [29]. The construction of this map placed *Phycomyces* as the model for analysis of genetic segregation in the Mucoromycotina. For comparison, the next two best-studied species are *Rhizopus stolonifer* and *Mucor hiemalis* [30], [31]. In these species, only two and six segregating markers have been examined. In *M. hiemalis* most products from the zygospore are a single genotype. The limited information from these two species illustrates how little is known about genetics in fungi, other than for the Dikarya which is the monophyletic lineage that encompasses the Ascomycota and Basidiomycota.

A major research focus on *Phycomyces* is its light-sensing abilities. The photosensory response of the sporangiophores is one of the best-studied traits in *Phycomyces* [11], [32]. Analysis of this property included the generation of phototropism mutants via chemical mutagenesis, starting in the late 1960s [33], [34]. Two of these genes, *madA* and *madB*, have been identified by predicting that they would be homologs of the photoreceptor complex genes *wc-1* and *wc-2* defined originally from *Neurospora crassa* [35], [36]. An additional eight genes (*madC*-*madJ*) remain to be identified. Other mutants affect unidentified genes that control tropisms to gravity and wind, primary metabolism, the biosynthesis of β-carotene, spore germination, mating, and colony and sporangiophore morphology [37]. The primary goal of this project was to develop a genetic map covering the genome that could be used for map-based cloning to identify these unknown genes. At the same time, this map would serve additional purposes: to estimate the number of chromosomes in this species, provide a higher order genome scaffold assembly, and examine the genetic reduction process that occurs within the zygospore.

## Materials and Methods

### Strains

**Figure 1:**
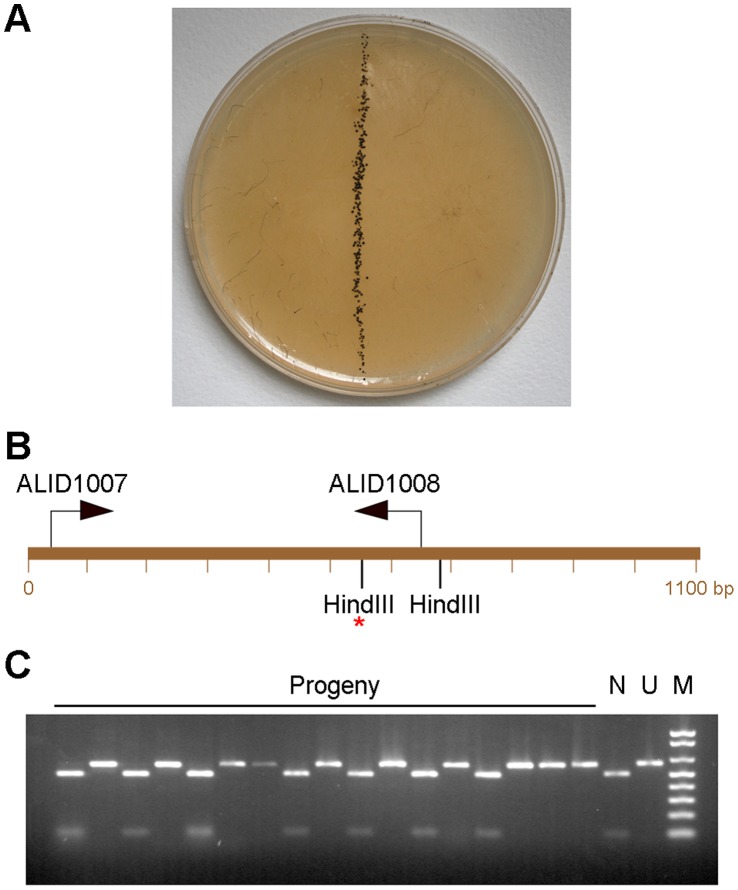
Three steps in generating the *Phycomyces* genetic map. **A.** Zygospores forming from a cross between UBC21 (+) and NRRL1555 (–) on V8 juice agar medium. The asexual sporangiophores have been removed from the plate for clarity. **B.** An example of primer design for PCR-RFLP markers. A restriction enzyme site polymorphism (HindIII *; scaffold 38 nucleotide position 361,161) between UBC21 and NRRL1555 was selected, and 550 bp on either side of the site examined by BLAST to ensure it was single copy in the genome and that it had no other restriction enzyme sites that would interfere with allele interpretation. **C.** HindIII digested PCR products resolved on a 1.2% agarose gel, from 17 progeny (a subset out of 121 total) and the parents NRRL1555 (N) and UBC21 (U) amplified with primers ALID1007-ALID1008. The markers (M) are the Invitrogen 1 kb+ ladder. The NRRL1555 allele generates 509 bp and 98 bp fragments. The UBC21 allele is uncut at 607 bp in size.

Wild type strains were NRRL1555 (mating/sex type –) and UBC21 (+). The mutant strains are listed in table 1, and details about the progeny from crosses provided in table 2. Strains were cultured on potato dextrose agar (Difco; Franklin Lakes, NJ, USA). Crosses were established on V8 medium (5% V8 juice, 4% agar, pH 6) (Fig. 1A). Zygospores were transferred to damp filter paper (2 mm thickness) in deep Petri dishes (2 cm total height) that were sealed with parafilm. Zygospores germinated 2–4 months later. For most crosses, the germspores from a single zygospore were resuspended in 10 µl H_2_O and heat shocked for 15 min at 45–48°C. The spore suspension was placed as a drop on a PDA plate, and the spores streaked to isolate single colonies. This was determined using a microscope prior to excision with a scalpel of the agar surrounding a single germinated cell. For a subset of zygospores, the spore suspension was plated as ten-fold serial dilutions on acidified minimal medium, that reduces colonial expansion [38]. Isolated colonies were subcultured onto PDA medium. 104 progeny from a cross between UBC21 and NRRL1555 had been generated previously [39], and 17 additional progeny were isolated to increase the population size. For scoring mating type, strains were crossed to NRRL1555 (–) or the congenic strain A56 (+) that had been generated by backcrossing the (+) allele into the NRRL1555 strain background [40]. For the analysis of mutant auxotrophic phenotypes, these were scored by the presence or absence of growth on yeast nitrogen base (YNB) medium. Resistance to 5-fluorouracil was assessed on medium supplemented with 0.25 mg/ml of this chemical (Acros Organics; NJ, USA).

**Table 1:** Strains used in this study. NG: isolated after mutagenesis with N-methyl-N′-nitro-N-nitrosoguanidine.

Name	Genotype	Parent and origin	Reference
NRRL1555	Wild type (–)		
UBC21	Wild type (+)		
A2	*furB* (–)	NRRL1555, spontaneous mutant	[27]
A34	*furA madD* (+)	B71×C149	[28]
A38	*carA furB* (–)	A2×C169	[28]
A849	*pyrF* (–)	NRRL1555+ NG	[26]
A851	*pyrF* (–)	NRRL1555+ NG	[26]
A859	*pyrG* (+)	A852×UBC21	[26]
A914	*lysA madI* (–)	A637×A845	[25]

**Table 2:** Progeny generated for genetic analysis. The parent named “progeny 82A” is derived from the cross between UBC21 x NRRL1555.

Parents	Number of progeny	Reference
UBC21 (+)×NRRL1555 (–)	121 (121 zygospores)	This study and [39]
UBC21 (+)×NRRL1555 (–)	176 (from 8 zygospores)	This study
A34 (+)×NRRL1555 (–)	40 (40 zygospores)	This study
UBC21 (+)×A2 (–)	10 (10 zygospores)	This study
A34 (+)×progeny 82A	15 (15 zygospores)	This study
UBC21 (+)×A914 (–)	45 (45 zygospores)	[49]

### Design of Molecular Markers

The development of PCR-based restriction fragment length polymorphism markers (PCR-RFLPs) underwent refinement during the course of the project. Genome sequencing information has been publicly available for NRRL1555 since 2006. The initial markers were designed based on the comparison of DNA sequences of a small genomic library constructed from strain UBC21 with the sequence of strain NRRL1555 [39]. In several cases these markers required restriction enzymes with low efficiency in reactions that included PCR components. In other cases, the markers amplified an additional product. Both scenarios complicated interpretation of the alleles. Subsequently, strain UBC21 was sequenced with Illumina technology by the US Department of Energy Joint Genome Institute. Polymorphic single nucleotides (SNPs) were incorporated into the genome sequencing browser, including an assignment of SNPs that change recognition sites for commonly-used restriction enzymes.

The majority of the molecular markers used amplification by PCR, and the alleles were defined by restriction digests of the PCR products. Prior to selection, 550–650 bp on either side of a polymorphic site were examined for additional restriction enzyme sites that could confound scoring of alleles after restriction digestion, and for copy number via BLASTn of the *Phycomyces* genome. 18–20 oligonucleotide primers were designed to amplify suitable regions. The sequences of the primers and their positions on the scaffolds of the *Phycomyces* genome release versions 1.1 and 2.0 are provided in table S1.

### Amplification of Molecular Markers

Polymorphic regions were amplified by PCR using rTaq and ExTaq DNA polymerase (Takara, Otsu, Shiga, Japan). The conditions were 2 min 94°C, then 32 cycles of 20 s 94°C, 20 s 52–55°C, 1 min 72°C, and a final extension of 5 min at 72°C. PCR products were digested using the appropriate restriction enzyme (New England BioLabs, Ipswich, MA, USA) and fragments resolved on 0.8–1.2% agarose gels prepared in Tris-acetate-EDTA (TAE) buffer. Optimized conditions used 15 µl PCR volumes and the digestion of the PCR after amplification, directly in 25 µl reaction volumes. Exceptions to PCR-RFLPs are as follows. Primers for amplification of *sexM* and *sexP* produce a product specific for each sex type. Primers ai814-ai861, ai1070-ai1071 and ai904-ai905 generate amplicons of differing size that were resolved on 1.4–2.0% agarose gels. Primer set ai990–ai998 produces amplicons of different size that were resolved on 20% polyacrylamide Tris-borate-EDTA gels.

### Identification of Genes

The genes *furA*, *lysA*, *nicA*, *pyrF* and *pyrG* were predicted based on previous biochemical or phenotypic evidence, and on equivalent functions in other fungi. Homologs were identified in the genome sequence database, the genes amplified from mutant strains and sequenced (primer details are in table S2). Primers for *lysA* were ALID0194–ALID202. Primers for *furA* were ai857–ai858. Primers for *pyrF* were ALID0449–450. Primers for *pyrG* were ALID0035–ALID0036. Sequences were compared to the wild type sequence from the NRRL1555 genome by BLASTn.

### Construction of Linkage Groups and Recombination Frequency Measurements

Allele information was recorded in Microsoft Excel (dataset S1) and exported to JoinMap 4.0 software [41]. Analyses utilized the multiple parameters available in the software. Based on the linkage groups and the maps obtained when compared to recombination frequencies between markers calculated manually, maximum likelihood methods were applied. Linkage between markers was first assessed by independence log of the odds (LOD). Recombination frequencies were calculated between markers in each linkage group assigned LOD 3 or higher. Linkage data were redrawn to scale in Canvas software for comparison to the physical distances between markers.

## Results

### A New Genetic Map of *Phycomyces* Resolves Nine to Twelve Linkage Groups

**Figure 2:**
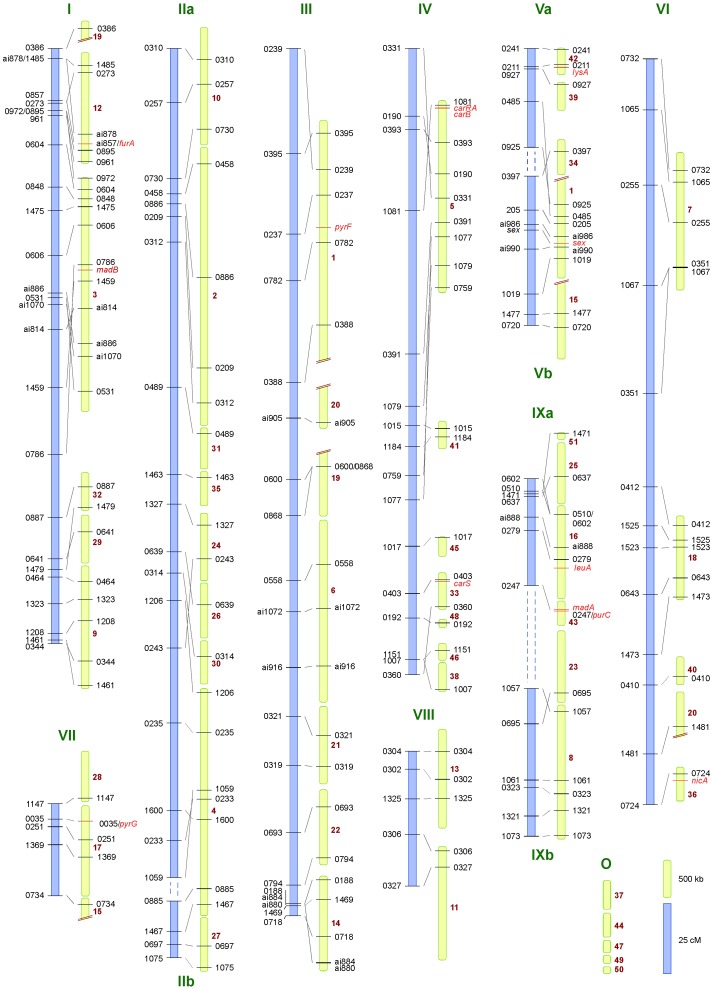
Alignment between genetic (left, blue) and physical (right, yellow) maps of *P. blakesleeanus*. Nine linkage groups are resolved at LOD score 3 and 12 at LOD score 5: the differences are indicated by dashed lines (these distances not to scale). Linkage groups are indicated in Roman numerals. Positions of the markers are indicated by the four digits of one of the two ALID primers or the ai### primer used. Positions of known genes are marked on the physical map in red. Scaffold numbers (red) are for genome sequence version 2.0. O = the five “orphan” scaffolds without molecular markers so not assigned to a linkage group. Four scaffolds are split (represented by two diagonal red lines) in forming linkage groups.

A set of PCR-RFLP markers was used to develop a genetic map from a cross between two wild type strains of *Phycomyces blakesleeanus*. 121 progeny were isolated, one from each of 121 zygospores of a cross between strains UBC21 (+) and NRRL1555 (–). Each progeny represents one of the outcomes of a fusion between the parents and the genetic reduction event from one zygospore (Fig. 1A). Markers were designed to the ends of each of the larger scaffolds in genome release version 1.1, then to the 50 largest scaffolds in version 2.0. Fig. 1B illustrates an example for the design of two primers, ALID1007–ALID1008, to amplify a region containing a HindIII site present in strain NRRL1555 and absent in strain UBC21. Five scaffolds (in version 2.0) did not have suitable locations for the design of markers, so are represented as the orphans (O) in Figure 2.

The markers were amplified by PCR from the 121 progeny, digested with restriction enzyme, and alleles scored by resolving the DNA on agarose gels (Fig. 1C). Four markers used size differences between the alleles, while the *sex* locus produces unique amplicons for each sex. This information (dataset S1) was imported into JoinMap 4.0 software for analysis of allele frequencies in the progeny and linkage between the markers.

The data were first examined for skews in allele distribution in the progeny. Progeny 53A, 66A and 113A had a skew in the number of alleles from the UBC21 parent. Progeny 99A and 138A amplified two alleles across multiple linkage groups, suggesting that these are either diploid or heterokaryons. This was also supported phenotypically in progeny 99A and 138A, by their production of pseudophore structures that are a hallmark of strains expressing both *sexM* and *sexP* genes. These five progeny were omitted from subsequent linkage analysis.

The remaining 116 progeny were analyzed for independence log of odds (with maximum likelihood) to test for potential linkage between the markers. The analysis revealed nine linkage groups with support of LOD 3. These scores compare the probability of linkage between markers compared to obtaining the data by chance, expressed as logarithm base 10. LOD 3 means 1/1000 that the results obtained occur by chance. At LOD scores of 4 or 5, three groups supported at LOD 3 were split, giving 12 linkage groups in total. The recombination frequencies between the markers on each of the 12 linkage groups were calculated, generating a map summing 1583.4 cM. The smallest linkage group was four markers over 20.2 cM and the largest linkage group 20 markers over 308.6 cM. The linkage groups are currently numbered I – IX based on the LOD 3 values. For the three linkage groups that would be split at LOD 5, these are further distinguished as IIa and IIb, Va and Vb, and IXa and IXb. The numbering system is arbitrary. Ideally the chromosomes should be numbered in sequential order of size: the data and state of the genome sequence preclude such conclusions at this stage. The relationship between the nine and 12 linkage groups are marked on figure 2 as dashed lines.

The linkage groups provide the opportunity to estimate the average recombination frequency over the genome, which reveals 1 cM equivalent to 33.2 kb. The value is calculated by summing the sizes of the scaffolds covered by markers (52.5 Mb) divided by the total cM. This number includes the DNA regions at the ends of the scaffolds, and could be adjusted in future by inclusion of other scaffolds, more accurate resolution of the amount of repetitive element DNA in the genome, or by adding more markers. Furthermore, for markers separated by long distances there are opportunities for double recombination events over the intervening DNA, skewing the estimate of an average frequency across the genome.

### The *Phycomyces* Genetic Map Correlates with the Physical Map

The genetic map and physical distances between the markers were compared (Fig. 2). The comparison reveals a range of recombination frequencies across the genome. Both maps are largely overlapping. Discrepancies between genetic and physical maps are located on scaffolds 1, 15, 19 and 20, where markers are found on more than one linkage group (LG). In addition, regions in which the linkage data suggest a possible inversion in the genome sequence assembly are found on LG I/scaffold 3 and LG IV/scaffold 5. These differences likely reflect the challenges of genome sequence assembly caused by repetitive elements in the genome, in that several of these breaks not present in the version 1.1 genome assembly.

### Identification of Genes to Anchor the New Molecular Marker Map

**Figure 3:**
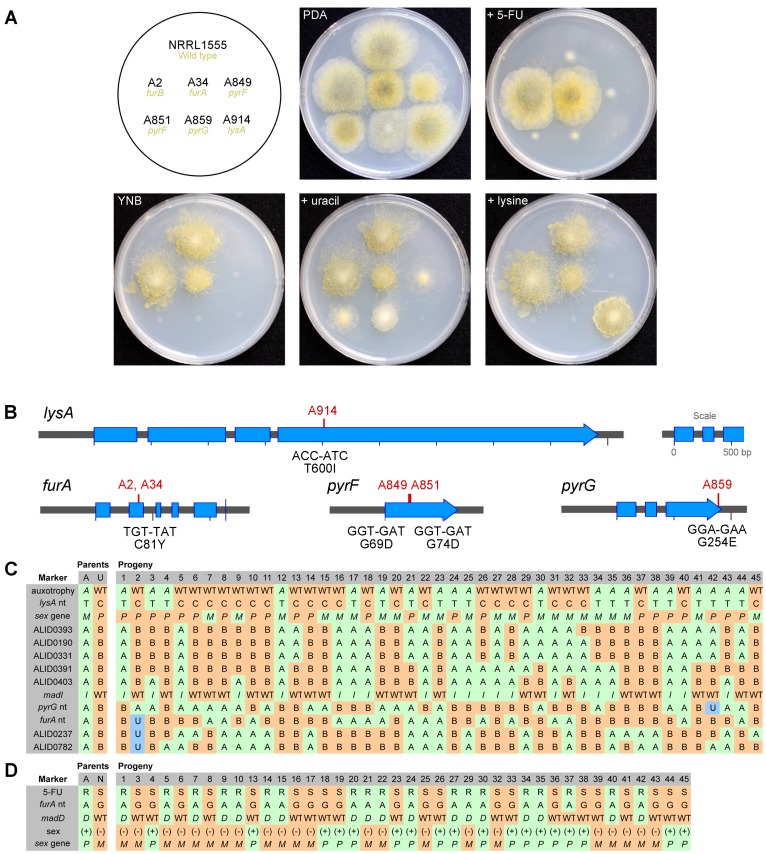
Mutations identified in strains of *Phycomyces*. A. Phenotypes on potato dextrose agar (PDA), PDA +250 mg/L 5-fluorouracil, YNB minimal medium, and YNB supplemented with uracil (20 mg/L) or lysine (30 mg/L) after three days growth at room temperature. The PDA was supplemented with uracil to support growth of the *pyrF* and *pyrG* mutants. **B.** The positions (red lines and strain names) and nature (codons and amino acid residue substitutions) of the mutations in the corresponding genes in these strains. **C.** Segregation data for *lysA*. Parents were A914 (A) and for UBC21 (U). Alleles are: for auxotrophy are either *A* for *lysA* or WT for wild type, the C or T nucleotide in the *lysA* gene, for PCR-RFLPs A for NRRL1555 and B UBC21, the *sex* gene is either *sexM* (*M*) or *sexP* (*P*), and the phototropism phenotype *I* for *madI* or WT for wild type. Progeny with both alleles are designated U and shaded blue and nt indicates a genotypic (rather than phenotypic) marker. **D.** Segregation data for *furA*. Parents were A34 (A) and NRRL1555 (N). Markers are: resistant (R) or sensitive (S) to 5-fluorouracil, the A or G nucleotide in the *furA* gene, *madD* (*D*) or wild type (WT) for phototropism, the sex phenotype is either (+) or (–) and equivalent PCR analysis for the *sexM* (*M*) or *sexP* (*P*) gene.

Eight *Phycomyces* genes have been mapped, which enables the incorporation of the previous genetics data with the new RFLP molecular marker map. In order to further strengthen these ties, additional genes with segregation information were sought. Predictions were made for *lysA*, *furA*, *nicA*, *pyrF* and *pyrG* based on the phenotypes of these mutant strains. Candidate genes were amplified from the equivalent mutants and sequenced. The nicotinic acid mutants of *Phycomyces* are under separate investigation (Alcalde, Idnurm and Cerdá-Olmedo, unpublished data), so are not discussed further here. Point mutations were found in all five genes, each causing a predicted amino acid substitution in a residue highly conserved in fungal homologs, and consistent with the phenotypes of the strains (Fig. 3A, B). To strengthen the evidence of the mutations causing the phenotypes for the two lesser characterized genes, genetic linkage was established for the point mutations in *furA* and *lysA* in crosses to wild type strains of the opposite mating type (Fig. 3C, 3D).

For *lysA*, 45 progeny from a cross between strains UBC21 (+) and A914 (*lysA madI* –) were inoculated onto yeast nitrogen base medium without amino acid supplements to score for the lysine auxotrophy. The *lysA* gene was amplified and sequenced from 45 of these progeny. 25 progeny were wild type and all had the wild type *lysA* sequence. 20 progeny were lysine auxotrophs and had the C-T mutation in the *lysA* gene. That is, the *lysA* candidate gene co-segregates with the mutant phenotype. Other markers, like a mutation affecting phototropism, segregated independently (Fig. 3C). The *lysA* gene and mating type were previously reported to be linked [28]. The *sex* alleles of the progeny were determined by PCR amplification. In this cross no evidence of linkage was observed between *lysA* and *sex* (22/45 or 48.9% recombinants).

For *furA*, 40 progeny were generated from a cross between strains A34 (*furA madD* +) and NRRL1555 (–), and the *furA* gene sequenced and phenotype scored on media containing 5-fluorouracil (5-FU). The *furA* mutation confers resistance to this chemical. 19 progeny were able to grow on 5-FU, and all 19 contained the mutation in *furA*. 21 progeny were unable to grow on 5-FU, and all 21 contained the wild type *furA* allele. A34 also carries a mutation in the *madD* gene required for phototropic response. The phototropism mutation and resistance to 5-FU were tightly linked, consistent with the previous segregation data for these two genes [27], [28]. The mating type was determined both by phenotype and by PCR amplification of the *sex* alleles, showing independent assortment of the *sex* locus with the *furA* and *madD* genes.

The *furB* gene was also sought. Like *furA*, mutation of *furB* confers resistance to 5-FU and *furB* is linked to *madD* [28]. Initially using genome version 1.1, scaffold 11 where *furA* is located was examined, but no obvious candidates were present. In the mapping data from the UBC21×NRRL1555 cross, scaffold 45 is linked to scaffold 11: both scaffolds are part of scaffold 3 in genome release version 2.0. This region contains a gene (JGI ID 163361) encoding a protein similar to xanthine-guanine phosphoribosyltransferases Hpt1 and Xpt1 of *S. cerevisiae*, making it a candidate for *furB*. However, amplification and sequencing of the gene from strain A2 (*furB*), provided from two sources, did not reveal any mutations. Finally, sequencing the *furA* gene from the *furB* strain A2 and a derived strain A38 revealed that the identical mutation in the gene was present as seen in the *furA* mutant strain A34, suggesting that *furA* and *furB* are identical.

**Figure 4:**
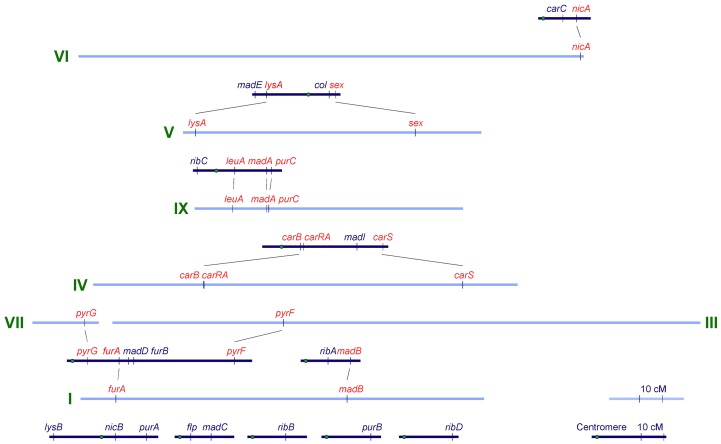
Comparison between genetic maps of *Phycomyces*. The previous mapping data is dark blue, redrawn from reference [29]. The positions of the genes in the new RFLP map (pale blue) are based on the locations of the closest markers to the gene. Five additional linkage groups were established in previous studies and remain to be anchored to the RFLP-based map.

With the identification of the genes involved in primary metabolism, their positions in new map were compared to the previous mapping data (Fig. 4). Further, SNPs were identified between the NRRL1555 and UBC21 alleles of *furA* and *pyrG*. Markers were designed to amplify these regions, and the alleles scored in the 121 progeny of the cross between NRRL1555 and UBC21, enabling exact correlation between molecular and phenotypic markers. This comparison shows consistency with the maps made from crosses between mutant strains compared to this crossing system between two wild type strains. One exception is the linkage group previously defined for *pyrG*, *furA*, *madD*, *furB* and *pyrF*, which are split in the UBC21×NRRL1555 data into three separate linkage groups, and the *furA*, *pyrF* and *pyrG* genes are also located on three different scaffolds in the genome sequence.

The lack of genetic linkage between *furA-madD* with the two *pyr* genes in the UBC21×NRRL1555 cross was further explored in three additional crosses (Table 2). The *furA* and *pyrG* genes were amplified directly, and the two markers that flank *pyrF* were amplified. No linkage was observed for these markers for the progeny of UBC21×A914, A2×UBC21, or A34×progeny 82A derived from the cross between UBC21×NRRL1555 (Fig. 3C and dataset S2). Summing all progeny, the percentage of recombinants between *furA* and *pyrF* is 46%–47%; *furA* and *pyrG* is 49%, and *pyrF* and *pyrG* is 48–50%.

### Irregular Gene Reduction Events Occur in Individual Zygospores

Previous analyses using phenotypic markers suggested that the reduction process in zygospores cannot be perfect meiosis. To examine this subject further, 176 progeny were isolated from eight single zygospores. Markers on each of the nine linkage groups (LOD score 3) were amplified and alleles assigned to the progeny (dataset S3). The expectation from meiosis is that each marker should be represented by each allele at equal frequency, and the markers on separate chromosomes segregate independently. Further, four genotypes should be observed per zygospore. Of the progeny from the eight zygospores examined only one produced four different genotypes. The progeny from the other zygospores provided evidence for amplification of one or two genotypes, while zygospore 1 yield progeny with markers all from strain UBC21. Furthermore, all eight zygospores produced progeny in which at least one allele only was present, rather than the expected 50∶50 ratio. This result is consistent with previous observations on skews from normal proportions for crosses between UBC21 and NRRL1555 [42].

**Figure 5:**
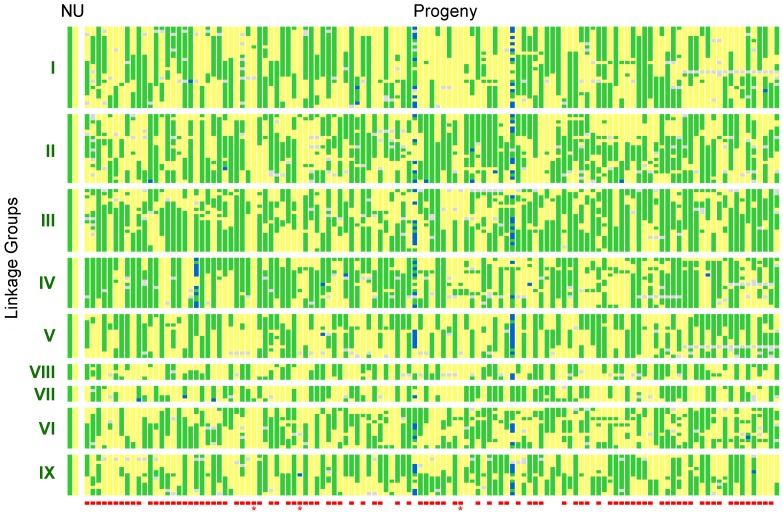
Graphical representation of allele segregation and recombination in 121 progeny. Each allele is colored green if from NRRL1555 (N), yellow if from UBC21 (U), blue if both, and grey if missing or ambiguous. The markers are divided into each linkage group (LOD score 3). The red boxes indicate progeny with one or more linkage groups with no recombination between the two parents (excluding the two smallest, VII and VIII), with the three progeny that inherited alleles primarily from UBC21 marked with *.

Based on the information of analysis from the eight zygospores, the data set of 121 progeny was re-examined for similar evidence of non-Mendelian reduction events. To be stringent, the data analyzed were the nine linkage groups supported at LOD 3 with the exclusion of the two smallest groups of five markers each (LG VII and LG VIII). There are 94 strains in which one or more linkage groups had markers from only one parent (Fig. 5, dataset S1). On the other hand, there is no progeny in which each linkage group showed segregation as a unit from each parent, although three of the progeny had most alleles derived from one parent.

## Discussion

Mendelian genetic analysis is a powerful experimental tool available for research on eukaryotic organisms, and includes unique capabilities to address specific aspects about the behavior of chromosomes. Map-based cloning has led to the identification of genes to understand their functions in numerous eukaryotes. The ability to perform crosses is particularly important in organisms that have not been successfully transformed. The primary aim of this study was to generate a genetic map that could be used as the framework for positional cloning of genes in the fungus *Phycomyces blakesleeanus*. Hundreds of mutants of this fungus have been isolated [37], yet due largely to the inability to transform DNA into this species only eight of those mutated genes have been identified. Secondary to this aim was to revisit the questions surrounding evidence for a sexual cycle in this fungus and, by extension, the Mucoromycotina.

We performed more than 16,000 PCRs to assign RFLP markers to 121 progeny from the cross between UBC21 (+) and NRRL1555 (–). The results show that recombination and crossing over occurs across the genome (Fig. 2; Fig. 5; dataset S1). This is an important observation for two reasons. First, cross over frequencies are one indicator of meiotic vs. mitotic recombination, discussed in more detail below. Second, recombination occurs across all linkage groups/chromosomes. Strain UBC21 has been recommended for genetic analysis since the 1970s. It has a short zygospore germination time (∼two months) when crossed with the laboratory standard strain NRRL1555, and is highly polymorphic with respect to NRRL1555 in which most mutants have been isolated. However, this divergence and the segregation ratios in progeny from crosses using this strain [40] were a concern in implementing it for map-based cloning. These segregation data suggest that this strain is suitable for map-based identification of genes, and may combine meiotic reduction with a proportion of non-recombinational transmission of the chromosomes, that together that would aid mapping.

Recombination rates within chromosomes of fungi vary between species. The *Phycomyces* estimate at 33.2 kb per cM is comparable to other sexual species. For example, this rate is the same as the mushroom-forming species *Coprinopsis cinerea* which is also 33 kb/cM [43] or plant pathogen *Gibberella zeae* which is 32 kb/cM [44]. *C. cinerea* is a model for meiosis in the basidiomycetes. *G. zeae* is a homothallic ascomycete whose ascospores are one form of inoculum during the disease cycle. Thus, the recombination frequency in the Mucoromycotina species *Phycomyces* is at a level observed in other fungi with well-established meiotic cycles.

The segregation data acquired here differ from the two previous approaches used for isolation of progeny of *Phycomyces*. One approach takes individual zygospores and scores all or many progeny derived from them. This has the power to map additional features of the *Phycomyces* genome, such as centromeres [14]. The second approach has been to pool the products from multiple zygospores, and treat the mix as in a random spore analysis. Our mapping approach used a single progeny taken from a single zygospore. This offers advantages by reducing physical manipulations or the analysis of large numbers of progeny.

Genes that have been sequenced and also mapped based on mutant phenotypes in *Phycomyces* are: *carRA* and *carB* that control biosynthesis of β-carotene [45], [46]; the *sex* mating type locus [39]; *madA* and *madB* that encode a blue light photoreceptor complex [35], [36]; *leuA* and *purC* that are linked auxotrophic markers [47]; *pyrG* [26], [48]; and recently *carS* for cleavage of β-carotene [49], [50]. Knowledge of other genes would help link the two maps more thoroughly. We identified base pair substitution mutations in strains carrying *pyrF* and *pyrG* mutations (Fig. 3). We also identified mutations within *lysA* and *furA*. *lysA* bears a mutation within a gene encoding α-aminoadipate reductase, the fifth step in the biosynthesis of lysine. *furA* bears a mutation within a gene encoding uracil phosphoribosyltransferase, required for salvage of pyrimidines. *nicA* was predicted to encode quinolinate phosphoribosyltransferase, the final step in the *de novo* biosynthesis of nicotinic acid from tryptophan [15]. A point mutation was identified in this homolog in a *nicA* mutant (Alcalde, Idnurm and Cerdá-Olmedo, unpublished). Genetic segregation analysis of progeny from crosses between the *lysA* and *furA* mutants with a wild type parent show perfect co-segregation of the mutations and the corresponding phenotypes (Fig. 3C, D).

The new genetic map was aligned with the genetic map generated previously (Fig. 4). Overall, there was strong congruence between previous and current segregation data. This includes linkage between markers near *purC*, *leuA* and *madA*; *lysA* and *sex*; *furA* and *madD*. One exception was the *pyrF* and *pyrG* genes that were previously mapped to the same linkage group as *furA*, *furB* and *madD* [26]. In our analysis, both the *pyrF* and *pyrG* genes are unlinked to these genes. The reason behind this discrepancy is unknown for *pyrG*: the difference could be explained by a chromosomal rearrangement in the *pyrG* mutant strain used to establish the linkage. In the previous crosses *pyrF* was distant from the *furA*, *madD* and *furB* cluster at 39, 42.5 and 47 cM away, depending on the marker and progeny characterization used. These distances are not far from that expected for unlinked markers. Direct comparisons between the two mapping approaches should be used with caution since different methods were used to generate the progeny and calculate recombination distances. Figure 4 also shows mapped genes defined by mutant phenotypes that are yet to be identified: comparison of the maps could prioritize the targets for these unknown genes.

Recombination and meiosis in *Phycomyces* have been topics of investigation since Burgeff’s studies in the 1910s, and aided by phenotypic markers since the 1970s. Despite being the most amenable species for genetics in the Mucoromycotina, the events during a cross remain unclear, even with independent Mendelian analyses. For instance, Mehta and Cerdá-Olmedo point out that their data and other segregation ratios are inconsistent with the model of one diploid nucleus undergoing a meiotic reduction [17], and that the evidence for meiosis in *Phycomyces* is scant. This is a valid point, although it is worth stressing that by similar criteria the evidence for meiosis is absent for most eukaryotes species, including many fungi.

To address the question of meiosis in *Phycomyces* further, we examined the evidence of crossing over on the establishing linkage groups. At least one cross-over event per chromosome is considered to stabilize chromosomes during meiosis [51]. Thus, the absence of recombination over a linkage group in one of the progeny may reflect nondisjunction of those chromosomes. We examined the UBC21 x NRRL1555 progeny set to look for examples of chromosomes with no recombination between markers. For this analysis, we used the map with nine linkage groups and excluded the two smallest linkage groups from the analysis because of their small number of markers. Overall, this gives the most stringent estimate from the available data, although it is not possible to exclude situations for centromeres positioned at the telomeric ends of the chromosomes. 94 progeny had one or more chromosomes that had all alleles from either UBC21 or NRRL1555. Together with the two diploid or heterokaryotic strains, 79% have this pattern of segregation (Fig. 5). However, with the three exceptions in which the progeny were primarily the UBC21 genotype, there were no examples in which every linkage group had been contributed into the progeny without recombination.

Our data cannot fully resolve the nature of the genetic reduction event(s) within the *Phycomyces* zygospore. These cells do demonstrate recombination, but also suggest the process is not “perfect” meiosis, as has been noted previously. The germinated zygospores compatible with one meiosis in a previous four-factor cross was about 78% [14]. We favor a hypothesis that the process relies on meiosis, and that it is influenced by poor or mistimed aligned of chromosomes during meiosis I. This is based on the relatively high rates of recombination (i.e. 33.2 kb per cM) observed in this cross, which is considered poor for marker segregation compared to crosses between strains with a higher proportion of shared genetic material [40]. Either *Phycomyces* undergoes meiosis or has a mitotic recombination frequency orders of magnitude greater than other eukaryotes. The second piece of evidence for meiosis is the *Phycomyces sex* locus, that resembles sex-determining regions in both fungi and animals. The *sexM* and *sexP* genes are within idiomorphic regions of DNA surrounded by conserved DNA sequence [52]. Third, genes encoding proteins with dedicated roles in meiosis [53], such as homologs of Dmc1, Spo11, Hop2, Mnd1, Msh4/Msh5, are found in the *Phycomyces* genome. Thus, the recombination frequency we observe, at the same level seen in other fungi, provides an experimental piece of evidence for meiosis in *Phycomyces*, and by extension the potential for meiosis broadly in the order Mucorales.

A caveat in interpreting the segregation data to assess the presence of meiosis in *Phycomyces* is that crosses between UBC21 and NRRL1555 may not be representative of a normal interaction between wild strains of *Phycomyces*. The geographical origin and year of isolation of both strains is unknown beyond culture collection acquisition. Improvement in “tetrad” frequencies increases with introgression of the genetic background [40]. For other crosses, the observed genetic ratios that are skewed from those expected after meiosis could reflect fitness defects for the auxotrophic strains used. In the case of segregation in the β-carotene mutants, it is possible that pheromone signaling plays a role after the fusion of the two parents. In the Mucoromycotina the sexual pheromones are produced by cleavage of β-carotene and subsequent modifications [54]. Pheromones are required for the sexual cycle after cell fusion in some basidiomycetes [55], [56] and ascomycetes [57].

The reliance on sexual reproduction for continuation of our species, and the deleterious effects when meiosis goes wrong, lead to a bias in interpreting this process. In the fungi it is becoming increasingly clear that some sloppiness in chromosomal segregation during mitosis, and presumably during meiosis, is advantageous by contributing to antifungal drug resistance and pathogenicity (e.g. see references [58], [59], [60]). The long dormancy period in the *Phycomyces* zygospore provides an advantage for dispersal over time and place, as well as resistance to environmental stresses. Any process that enables the production of another generation from the genetic material stored within the zygospore would suffice in most cases to create recombinant strains, at a later time point and distant place than when and where the original parental genotypes existed.

In the intervening time since the first progeny were isolated and markers were developed to create this genetic map (more than seven years), genome sequencing and other technologies have undergone rapid advances. More efficient technology is now available that can be applied to mapping. For example, a high-resolution map could be generated from the genome sequences of each progeny. For mapping mutations to parts of the chromosome, bulked segregant analysis and genome resequencing could be used, as has been successful for mapping in *Neurospora crassa* [61]. We have already applied these PCR-RFLPs for map-based identication of four genes, *carS*, *leuA*, *madC* and *madI*, and narrowing the DNA regions for others ([47], [49] and unpublished data). The identification of the *furA*, *lysA* and *nicA* genes adjacent to *madD*, *madE* and *carC* genes, respectively, should facilitate the identification of genes required for phototropism and biosynthesis of β-carotene. Thus, positional cloning is a viable method for gene identification in *Phycomyces*.

## Supporting Information

Table S1 **Primer information for molecular markers.**
(DOC)

Table S2 **Primers used to amplify and sequence** ***furA*** **,** ***lysA*** **,** ***pyrF*** **and** ***pyrG*** **.**
(DOC)

Dataset S1 **Allele details for progeny from the crosses between UBC21×NRRL1555.** A, allele from NRRL1555; B, allele from UBC21; U, both alleles or ambiguous. Grey boxes indicate weak or failed PCR reactions.
(XLSX)

Dataset S2 **Allele segregation in progeny isolated from crosses between UBC21×A2 and A34×progeny 82A.** Strains with alleles from both parents are designated U and shaded blue.
(XLS)

Dataset S3 **Allele details from progeny isolated from eight zygospores of crosses between UBC21×NRRL1555.** A, allele from NRRL1555; B, allele from UBC21; U, both alleles or ambiguous.
(XLS)
