# Supplementary material for: A New Genetic Linkage Map of the Zygomycete Fungus Phycomyces blakesleeanus
Source: PLoS One. 2013 Mar 14;8(3):e58931. doi: 10.1371/journal.pone.0058931 (PMC3597544; doi:10.1371/journal.pone.0058931)
Supplement: Table S1 — Primer information for molecular markers. (DOC) [file pone.0058931.s001.doc]

**Supplemental table 1.** Primers used as molecular markers and the position of the polymorphism they detect in the two *Phycomyces* genome releases at JGI.

| **Name** | **5'-3' sequence** | **Genome v 1** | **Genome v 2** | **Enzyme** |
| --- | --- | --- | --- | --- |
|  |  |  |  |  |
| ALID0035 | ATTCTTTACTTTAGCTTCG | 8 @ 1205403 | 17 @ 205903 | BstZ17I |
| ALID0069 | AATCTACCTTGAGTACGC |
| ALID0188 | TCCTGAAATGAAGAGACG | 72 @ 97129 | 14 @ 49517 | EcoRV |
| ALID0189 | TATATTCTCGGAGGTCTG |
| ALID0190 | ACCAGCGCTGGACAACAC | 6 @ 785931 | 5 @ 977527 | NdeI |
| ALID0191 | TATTCCGCAAACTGATCC |
| ALID0192 | TGGTGCCTACCAGCTTCG | 77 @ 57969 | 48 @ 49363 | NdeI |
| ALID0193 | GATTTCTTAGTCATTGTC |
| ALID0205 | CGAACAGAACTACAGACG | 41 @ 35906 | 1 @ 3721421 | RsaI |
| ALID0206 | TACAAGTTTCCCACTGCC |
| ALID0209 | AATGTCTAAACTTACAGG | 1 @ 756545 | 2 @ 765824 | HindIII |
| ALID0210 | AGAAGATTTGTCTCCCAG |
| ALID0211 | GCCGAGAAGACTGAGTTC | 53 @ 47782 | 42 @ 222131 | EcoRI |
| ALID0212 | GAGTGTCATGTCTAATAC |
| ALID0233 | CACGCAAGCGATCATAGG | 3 @ 1461054 | 4 @ 1474019 | BamHI |
| ALID0234 | ACACAGTTTGTCTCTTGC |
| ALID0235 | CAATGGAGTGATGCTGAG | 3 @ 576060 | 4 @ 587227 | KpnI |
| ALID0236 | TTGCGACTTCAGAAAGTTG |
| ALID0237 | ATGATCGTCATGGTACAC | 4 @ 916674 | 1 @ 955002 | BamHI |
| ALID0238 | ATGTCGTCTATAATGTTG |
| ALID0239 | TGAACTGAAAATGAAGTC | 4 @ 580205 | 1 @ 616632 | HindIII |
| ALID0240 | TGATGCCCTTTACTATCG |
| ALID0241 | TTAAATGTTCTCAAGAGC | 53 @ 238951 | 42 @ 24073 | BamHI |
| ALID0242 | TCTTCCATTGATAGTTCC |
| ALID0243 | AATGAACTTGTTGGCTGG | 7 @ 567565 | 24 @ 291568 | BamHI |
| ALID0244 | TTTATGCCTCTAGCGCCC |
| ALID0247 | TCTTGCTGGTTACTTCCG | 36 @ 140027 | 43 @ 191403 | BamHI or PstI |
| ALID0248 | TCATCTTCATGGACATGG |
| ALID0251 | AGTTGAACTTACTTACGC | 8 @ 956232 | 17 @ 456078 | EcoRV |
| ALID0252 | TCTAGAACTAGAAGACTC |
| ALID0255 | GTGGAGAATTGTACACCC | 9 @ 606273 | 7 @ 931008 | KpnI |
| ALID0256 | TTCTTGATCTACTGAACG |
| ALID0257 | CAGAAGAAGTACAGATGC | 10 @ 546915 | 10 @ 809318 | EcoRV |
| ALID0258 | TATATGAGTTGTAGCGTC |
| ALID0273 | AACCGGATTCGTGGATTC | 11 @ 78790 | 12 @ 1208900 | EcoRI |
| ALID0274 | TCGATCGTAAAGACAGGG |
| ALID0279 | ATGCGCAGAAGACTTGAG | 15 @ 339189 | 16 @ 716502 | HindIII |
| ALID0280 | TAAAATACCTGGTGCTGG |
| ALID0302 | AGCCATTGAGATCCAAGC | 12 @ 467243 | 13 @ 668508 | PstI |
| ALID0303 | GACCAAAAGTCTACACAG |
| ALID0304 | TTATCAATATCAGCCAGC | 12 @ 839824 | 13 @ 296061 | BamHI |
| ALID0305 | CCCAGATGAAGTTGAGCG |
| ALID0306 | TATTACCTGGTAAAAGCC | 13 @ 87241 | 11 @ 61161 | EcoRV |
| ALID0307 | ACCACCGATTATACCATC |
| ALID0310 | ATAGAGATTATCTGCACC | 10 @ 872873 | 10 @ 1137818 | EcoRV |
| ALID0311 | GGGCCAGATCAATCTGTG |
| ALID0312 | TTAGCTATGGCAACACTC | 1 @ 299988 | 2 @ 300343 | EcoRV |
| ALID0313 | CAAGTTATCGAATGTCTG |
| ALID0314 | TGGCTCTTATTCAATGTG | 7 @ 1307797 | 30 @ 208958 | EcoRI |
| ALID0315 | ATCGGTGTATCTGATCTC |
| ALID0319 | TACTTTGGCTACATCATC | 14 @ 228140 | 21 @ 803404 | BamHI |
| ALID0320 | TCCAATCCATGAATACGG |
| ALID0321 | TAAGGTGTTTACCAGTGC | 14 @ 638653 | 21 @ 388932 | KpnI |
| ALID0322 | ACTCATGCTACACCTACG |
| ALID0323 | ACCAGGTACTTTGGCTGC | 20 @ 142653 | 8 @ 1183617 | HindIII |
| ALID0324 | GACACAGCATCAACACTG |
| ALID0327 | TGTGGTGATCGTGGAATC | 13 @ 299005 | 11 @ 278063 | HindIII |
| ALID0328 | AGAGTCTCCCTGTAGCTG |
| ALID0331 | TAACTTGATCTCTTCAGG | 6 @ 482278 | 5 @ 1301406 | EcoRV |
| ALID0332 | TTGGGAAACATCAGTATC |
| ALID0344 | AGACCTTGGCTGTCATGG | 8 @ 269586 | 9 @ 368941 | BamHI |
| ALID0345 | CTGCTTCTCCATTGAGGC |
| ALID0351 | GCTGATTGTGTGTCATTC | 9 @ 1196692 | 7 @ 1529591 | EcoRV |
| ALID0352 | GAGGGCGATGAATAAACC |
| ALID0360 | TGTGTCTAAGGATAACCC | 32 @ 431828 | 33 @ 36117 | BamHI |
| ALID0361 | GAGTTGATGCAAGCGAGG |
| ALID0386 | AGAAAAGGATTTGCTCGACC | 43 @ 98915 | 19 @ 105823 | BamHI |
| ALID0387 | GGAAAAAGTGGTGGTTCAGC |
| ALID0388 | TTCTAGATTGCATGGTTC | 4 @ 2633050 | 1 @ 2689017 | EcoRV |
| ALID0389 | TGGCTTACAATAAGATCC |
| ALID0391 | GCAAATATCTCTCAGTCG | 6 @ 155942 | 5 @ 1626543 | EcoRV |
| ALID0392 | TAGTCGAGGGCTCTTGTG |
| ALID0393 | CCCATTTCTTGTCCCTGTAG | 6 @ 1215290 | 5 @ 557248 | EcoRV |
| ALID0394 | CATTCACAAAATAGCACAGC |
| ALID0395 | ACATCGGGTACAATAGAC | 4 @ 95027 | 1 @ 130654 | NdeI |
| ALID0396 | CTTTGATGGCGTATCAGG |
| ALID0397 | CCATTTGTAGGGTGAAG | 47 @ 143449 | 34 @ 157232 | XhoI |
| ALID0398 | GCTAAATCAACAGAGTCC |
| ALID0403 | TGGACCGTTATGACCAGG | 32 @ 66900 | 33 @ 394124 | EcoRV |
| ALID0404 | TCTATAAGATGGCAGGTG |
| ALID0410 | GGTAAGTCTAAGTTTGAC | 67 @ 110127 | 40 @ 260327 | EcoRV |
| ALID0411 | GATTAGCATTCATACACC |
| ALID0412 | TAGAATTGAGCTGTCTGC | 35 @ 354976 | 18 @ 125307 | EcoRI |
| ALID0413 | AGAGGTGCTATGGCACAC |
| ALID0458 | AGATAAGTTCGTTGGCTC | 1 @ 3445767 | 2 @ 3488603 | HindIII |
| ALID0459 | AGTCATAGAATACGTGTG |
| ALID0464 | ATTGCCATTAAAGAAGCC | 19 @ 204319 | 9 @ 1428702 | EcoRI |
| ALID0465 | AATTCGTTAGAAGGAAGC |
| ALID0485 | AACGTTTGTGGCAAGTGG | 30 @ 252236 | 1 @ 3627299 | BamHI |
| ALID0486 | TTGCAAGGCGACGATCAG |
| ALID0489 | ATACTTGAGGCTTGGTGC | 18 @ 77208 | 31 @ 77241 | HindIII |
| ALID0490 | TGGTTACACGATAGTCGC |
| ALID0510 | TAATGGTGTGACATGGAC | 15 @ 919915 | 16 @ 113686 | EcoRV |
| ALID0511 | GCTGTAGGTAGTCGAGAC |
| ALID0531 | GTAGTCTTGCACTGTAGC | 5 @ 241231 | 3 @ 2851012 | EcoRI |
| ALID0532 | GACACATACAGACCTTCG |

| ALID0558 | TTCATTTCCAGCTGTCAG | 2 @ 1821852 | 6 @ 1837466 | EcoRI |
| --- | --- | --- | --- | --- |
| ALID0559 | TGAAAGGTATGCGTTGCC |
| ALID0600 | CCTGAACGACGAATCATC | 2 @ 2621645 | 19 @ 420023 | XhoI |
| ALID0601 | GCAACCACAAGATCTGAC |
| ALID0602 | TCAAGTACAGAAATCAGC | 15 @ 919915 | 16 @ 113686 | EcoRV |
| ALID0603 | AAGATTCAGCAAAGACAC |
| ALID0604 | ACCTTGGATCTCTGAACG | 45 @ 178553 | 3 @ 160981 | XbaI |
| ALID0605 | ATCTTGCCTCACCAAGAC |
| ALID0606 | ATCAGCAGCAAATCCAGC | 29 @ 290285 | 3 @ 633377 | NdeI |
| ALID0607 | TCAACTGACATACGAAGC |
| ALID0637 | TGTGGAAGCAAATCACCG | 25 @ 275064 | 25 @ 448339 | BamHI |
| ALID0638 | GTTGACCAGCAGTTGAGG |
| ALID0639 | CAAGAGGTGGACATTTGG | 26 @ 283735 | 26 @ 290207 | EcoRV |
| ALID0640 | GGCTGGTGCAAATTCTAC |
| ALID0641 | TGGCGAATTCAATGTCGG | 27 @ 207140 | 29 @ 225025 | XbaI |
| ALID0642 | CTGCCTACACCTTCAAGC |
| ALID0643 | AATACTATCCCTCCCGAG | 28 @ 282199 | 18 @ 828003 | XbaI |
| ALID0644 | TAAATCGTCATGTGGCTC |
| ALID0693 | CGCAACTTGTCTTGTGGC | 16 @ 769043 | 22 @ 232242 | HindIII |
| ALID0694 | ACATGGCACACAGACAGC |
| ALID0695 | CGTTGAAAGCATTAAGGG | 17 @ 822856 | 23 @ 828723 | HindIII |
| ALID0696 | ACTGCCTTTACCTTGGTC |
| ALID0697 | GTGAGGCTTTCTAGGACT | 22 @ 328423 | 27 @ 336199 | PstI |
| ALID0698 | TCATCCTACAACTTTGGC |
| ALID0718 | TGCAACCACGGCTGTCGAGC | 24 @ 56416 | 14 @ 808548 | BamHI |
| ALID0719 | TGCGCACCAATCTGATCAGGGA |
| ALID0720 | GGTCGTGGGCTTGATCGCGT | 31 @ 43746 | 15 @ 841172 | XhoI |
| ALID0721 | CCCTCCGGTCTCGACCCAGAA |
| ALID0724 | GACTCCGCAGCTTTGGGAGCA | 37 @ 83439 | 36 @ 88778 | XhoI |
| ALID0725 | AGCTCTTGCATTCACAGGCTTTCCAG |
| ALID0730 | GTTCGCAAGCCCACTGAGCC | 46 @ 99037 | 10 @ 208183 | HindIII |
| ALID0731 | ACAGCGAGAGCAGGACTGAAGGT |
| ALID0732 | AGCCGCATTGTTGTCATGGTCG | 48 @ 76596 | 7 @ 241138 | XbaI |
| ALID0733 | CAGCGTCCTGGCACTGGCAT |
| ALID0734 | ATCCACAGCCAAGCTCTCGGT | 49 @ 70344 | 15 @ 84628 | XbaI |
| ALID0735 | ACTGCAAATGCAAAGCCTCAGGT |
| ALID0759 | GAACAAGGAGATTGAAGACC | 21 @ 48534 | 5 @ 2498165 | XhoI |
| ALID0760 | TTGCTGCTCTAAAGCAGATG |
| ALID0782 | ACCCTGTCGTTGCTCGTTGGT | 4 @ 1537597 | 1 @ 1585926 | PstI |
| ALID0783 | AACTGCCGGAGATGCTCTAAGGA |
| ALID0786 | TGGCGCCAAAACCCAAATGCT | 5 @ 1946156 | 3 @ 1162594 | XhoI |
| ALID0787 | AGGCCTGTGTGCGATGCACTG |
| ALID0794 | ACGAAGCGAAACAGAGCTAC | 16 @ 91431 | 22 @ 912389 | XhoI |
| ALID0795 | GTCAGTTATGGATGAGTTCC |
| ALID0848 | GCAATAACGAATAGAGGGTACC | 45 @ 58324 | 3 @ 282508 | XbaI |
| ALID0849 | CTGACGGTGTTGCTTGGTAG |
| ALID0866 | GTCAATTCTATTCAACGCTC | 1 @ 1945977 | 2 @ 1971689 | EcoRI |
| ALID0867 | GGAATACTAGACTGATAGG |
| ALID0868 | AGAAGTCTTCCTGAACGACG | 2 @ 2621645 | 19 @ 420023 | XhoI |
| ALID0869 | TTCTTGGCAACCACAAGATC |
| ALID0885 | TCACTTCATGATCCTCTACG | 23 @ 255480 | 4 @ 2670063 | HindIII |
| ALID0886 | ATCCTTACGGCCTCAGATTG |
| ALID0887 | GCTACGTTGTTGAGGATGAG | 33 @ 297881 | 32 @ 313016 | HindIII |
| ALID0888 | AAGTAGAGTATCCTTCGTGG |
| ALID0895 | CAGTTCCACCCTTGCCACAC | 64 @ 174750 | 12 @ 173931 | PstI |
| ALID0896 | GTGACTACCAGCAGTTTCCAC |
| ALID0925 | GAACTATCGCCTGGTATTGG | 30 @ 96633 | 1 @ 3469899 | PstI |
| ALID0926 | GTACACCTCAATATTCTCTCC |
| ALID0927 | CCATCATAAGGGCAAGGTG | 30 @ 538812 | 39 @ 344514 | EcoRV |
| ALID0928 | GTAAATTTGTCCAGAGGTGG |
| ALID0961 | GACGAGAAAGAACAGTGTCC | 64 @ 30212 | 12 @ 22355 | NdeI |
| ALID0962 | CTTTGCTGTTGTTGCTGTAGC |
| ALID0972 | GCAGACGTTGAGAAACTAGG | 45 @ 328247 | 3 @ 6281 | EcoRV |
| ALID0973 | CTTGCCAGATGTTGTTGTTC |
| ALID1007 | GTACTGTACATCACGCCATGTG | 38 @ 37775 | 38 @ 361161 | HindIII |
| ALID1008 | TTGGCCAAAGTGAGGGGAGTGC |
| ALID1015 | ATACCTTATGATTCCCGATG | 44 @ 93163 | 41 @ 268336 | NdeI |
| ALID1016 | GTGTGTGTGAGAGATTTCAAG |
| ALID1017 | CTGGCTCGCTCAAGGTCGAG | 54 @ 246071 | 45 @ 9911 | XhoI |
| ALID1018 | AACTCAAGATCGTTATGCTG |
| ALID1019 | AGTTTCTCGGGTTCAGGGGCG | 56 @ 149177 | 1 @ 4190291 | EcoRI |
| ALID1020 | GGCAACCACAAAGATTG |
| ALID1057 | CAACAAGAATAGCACAGAAG | 3 @ 3137726 | 8 @ 90061 | XbaI |
| ALID1058 | TAATTCACCAGTGGCATAGC |
| ALID1059 | CAAGCAGTCGCCCATAAG | 3 @ 1339021 | 4 @ 1355567 | EcoRI |
| ALID1060 | ATGGGTCTTTTAGTCCAC |
| ALID1061 | CCCGTTATCTGGACTTCC | 3 @ 2209379 | 8 @ 1010829 | BamHI |
| ALID1062 | GTTGAGAAAGGACATGGACC |
| ALID1065 | TGCTGTGACTAGCGACTC | 9 @ 70989 | 7 @ 394002 | EcoRI |
| ALID1066 | AGATCTGCTGTCAAACGC |
| ALID1067 | TAGACCGCACATTAAATGTC | 9 @ 1206343 | 7 @ 1539242 | NdeI |
| ALID1068 | ACTGTAGTGCGAACAGCTTC |
| ALID1073 | GGGTTTGATCCGTTTACG | 20 @ 697119 | 8 @ 1740663 | HindIII |
| ALID1074 | TAATGTTTCAGATTGGCGTG |
| ALID1075 | AAATGTTGGGTCTACTTGTG | 22 @ 46724 | 27 @ 49241 | NdeI |
| ALID1076 | CTCTGATTCGGATCAAGGAG |
| ALID1077 | CTATTGTTATCATTCAGGAGG | 21 @ 724903 | 5 @ 1816625 | XbaI |
| ALID1078 | AATATCGCAGTTGCATTGAC |
| ALID1079 | AATCCGTGAGATAAGCACAG | 21 @ 343327 | 5 @ 2201014 | EcoRV |
| ALID1080 | TAAGATGTACATCGTCCTAC |
| ALID1081 | TATGCTCCTGCCAAACGTCC | 6 @ 1704583 | 5 @ 61007 | XbaI |
| ALID1082 | TTCCGTAGCAACAGTGCTGC |
| ALID1147 | GTGGCAGGCGAAGATTATAC | 50 @ 226876 | 28 @ 626711 | EcoRV |
| ALID1148 | CAAGGTTAGCGTAATACCAAC |
| ALID1151 | GTCCTGTGGGAAATACTAAG | 38 @ 191603 | 46 @ 94690 | PstI |
| ALID1152 | CAGAAACACCCATAGAGCTC |
| ALID1184 | TCGTGCTACCGTACAAACCCCT | 44 @ 205583 | 41 @ 155605 | NdeI |
| ALID1185 | ACCCCGGTCTAATTGGGGTCTAGA |

| ALID1206 | AGCAATAGTAGTAGTAGTGG | 3 @ 51989 | 4 @ 55762 | BamHI |
| --- | --- | --- | --- | --- |
| ALID1207 | CACTCATGGATGTAGAGACC |
| ALID1208 | TGTCTCTTGAATAACCGCTG | 19 @ 725870 | 9 @ 905750 | HindIII |
| ALID1209 | ACACTCTCCTTCTTAGGTGC |
| ALID1321 | CTCCCAACAATCTGATGTCG | 20 @ 364163 | 8 @ 1406375 | EcoRI |
| ALID1322 | ACAAGGTAATCTCCTGCTCG |
| ALID1323 | CATCACCTTGGATACAACGC | 19 @ 443940 | 9 @ 1188840 | EcoRV |
| ALID1324 | AGCTGTTCCTTGACGTAGCC |
| ALID1325 | GTAGCCTCTGTAAAGATCCG | 12 @ 214594 | 13 @ 924305 | XhoI |
| ALID1326 | TGTACAGAATGGTCACACGC |
| ALID1327 | ACCGAGTTCGTGATAACGTG | 7 @ 119977 | 24 @ 742922 | HindIII |
| ALID1328 | GCTTGTCCTGATGCTCATGC |
| ALID1369 | GCTGTTGTGTCTTCCACAAC | 8 @ 718981 | 17 @ 684315 | EcoRV |
| ALID1370 | AGACTATTGGTAATCAGGTC |
| ALID1459 | GATTACCACCTTTTGCACGC | 5 @ 1727917 | 3 @ 1381338 | KpnI |
| ALID1460 | CCGTTAGCACGACATCAAAC |
| ALID1461 | GGTATGCTGACTATCAGAG | 8 @ 593334 | 9 @ 44251 | PstI |
| ALID1462 | GTAATCACATCCCAAAAGC |
| ALID1463 | CACTGGATACGCGAGATAAC | 18 @ 613501 | 35 @ 75941 | EcoRV |
| ALID1464 | CCAATTGAAACCAGCCCTTG |
| ALID1467 | GATGTTGGCGTGTTATCTGG | 23 @ 37403 | 4 @ 2891627 | EcoRI |
| ALID1468 | CTGTTCCTCATGTGGTATGG |
| ALID1469 | GTTCCTCCTCAACAGTCTAG | 24 @ 556951 | 14 @ 311752 | HindIII |
| ALID1470 | CTTCCAGATACTGCTCAAGG |
| ALID1471 | GTCGAAGAATCGTATCTCTC | 25 @ 633362 | 51 @ 6520 | EcoRI |
| ALID1472 | CAGCGTACAAAAGAAGAAGC |
| ALID1473 | CCTACACACACACACAAGTC | 28 @ 37309 | 18 @ 1082238 | EcoRI |
| ALID1474 | GACCCTTTAGATCCTTTCGG |
| ALID1475 | CAAAGCCATCGGGTCTAATG | 29 @ 626604 | 3 @ 389296 | HindIII |
| ALID1476 | CCGTTATTTGTGCTCGTCTG |
| ALID1477 | CGAGTAGATTGTCCAGAAAC | 31 @ 210951 | 15 @ 657365 | EcoRI |
| ALID1478 | GCTGAATGACAGAGTGCATC |
| ALID1479 | GTCTCACCACTACTTCGAAC | 33 @ 32618 | 32 @ 38111 | NdeI |
| ALID1480 | GTGACGATTACAGGAAGTGC |
| ALID1481 | GCCAATCTTGTTTCTCTAGC | 34 @ 180527 | 20 @ 461478 | XbaI |
| ALID1482 | CGAACGTAGGATAGTTTTGC |
| ALID1485 | GTAGAAGGGTGATGGCATAC | 43 @ 337499 | 12 @ 1304885 | PstI |
| ALID1486 | CTGTCACAGATCCCACTATC |
| ALID1523 | GTGCTTGACCCATATCTTGG | 22 @ 696489 | 18 @ 30205 | HindIII |
| ALID1524 | CAATAGTAAAAGGGGGGGAG |
| ALID1525 | CAGTAGGAGCATAGAGCATG | 35 @ 157594 | 18 @ 323728 | HindIII |
| ALID1526 | CCATTCAGTCAAGTAGACGG |
| ALID1600 | GCAAGATTAGTGGAAGCCAG | 3 @ 1729393 | 4 @ 1746083 | HindIII |
| ALID1601 | CTTGAAAGCCAAAGTCCTCC |
| ai814 | TATATAAAAGAAGAAGCGGG | 5 @ 1364142 - 1364199 | 3 @ 1744272-1744329 | Size |
| ai861 | GATAACAGATCCGTAAGCTC |

| ai857 | TGCATTGCACGACTTTACCC | 11 @ 1023372 | 12 @ 260793 | TaqαI |
| --- | --- | --- | --- | --- |
| ai858 | ATACCGCTATCTATAAACGC |
| ai878 | CGATTGTATCGTTCTCAAGG | 11 @ 896129 | 12 @ 388129 | NdeI |
| ai879 | CTTCAGCCGACCATATTCC |
| ai880 | TATTACCTTTTTGCCGAC | 40 @ 109846 | 14 @ 1159546 | NdeI |
| ai881 | CCACCATTCCAATTTGGTAC |
| ai884 | TGTTTTGGTTATACTCCG | 40 @ 114291 | 14 @ 1155101 | BfaI |
| ai885 | AGGGAATTTAGAGCGAGG |
| ai886 | GTGGAGCTTTAGATTAGG | 5 @ 896531 | 3 @ 2213099 | MspI |
| ai887 | AGACACTCTCTGGGCCAC |
| ai888 | ACTTGTGGAAAATGCTTTAG | 15 @ 414089 - 480812 | 16 @ 559212  - 621795 | HpyCH4V |
| ai889 | AAATTGCCAGTCAAAATG |
| ai916 | AACCCTGACGATCCTTACGC | 2 @ 474932 | 6 @ 482368 | BamHI |
| ai917 | CCATTTCCTCAGATATGCAC |
| ai986 | TCCCTGAAATCAGCCACCAC | 41 @ 211594 | 1 @ 3897744 | MspI |
| ai1018 | TTCAATATCATACGCATACC |
| ai990 | ACAAGCTACAGAGATCAAAC | 41 @ ~348119 | 1 @ ~4036259 | Size |
| ai998 | ATCTCATCTTCATCTACGCC |
| ai1070 | GTCACGTTTAATTCTCCC | 5 @ ~723000 | 3 @ ~2385000 | Size |
| ai1071 | GATGTTGGGAGAGTTATC |
| ai1072 | TTAAAACCCAGATAGAGG | 2 @ 1234672 | 6 @ 1249285 | SspI |
| ai1073 | TGATTCAAAATGTTCAGG |
| ai904 | CCTAGTAGTCACGCCAAACG | 2 @ 3483314 | 20 @ 988389 | Size |
| ai905 | GAGAGTATTTATACTGTGGG |
| ai978 | CCTAGCATTCTATCAAGC | 41 @ 297810 - 298445 | 1 @ 3985605 -3986240 | *sexM* gene |
| ai978 | GTAGTCTTGTGAGGTAGC |
| ai950 | GCAAATGTGTACCGAAAGC | 41 @ ~297810 - 298445 | 1 @ ~3985605 -3986240 | *sexP* gene |
| ai951 | ATTATTACAAAAGTCCTGCC |
