# Supplementary material for: A New Genetic Linkage Map of the Zygomycete Fungus Phycomyces blakesleeanus
Source: PLoS One. 2013 Mar 14;8(3):e58931. doi: 10.1371/journal.pone.0058931 (PMC3597544; doi:10.1371/journal.pone.0058931)
Supplement: Table S2 — Primers used to amplify and sequence furA , lysA , pyrF and pyrG . (DOC) [file pone.0058931.s002.doc]

**Supplemental table 2.** Primers used for amplification and sequencing of genes in *Phycomyces*. F = forward and R = reverse primers used in PCR.

| **Name** | **5'-3' sequence** | **Gene** |
| --- | --- | --- |
|  |  |  |
| ai857 F | TGCATTGCACGACTTTACCC | *furA* |
| ai858 R | ATACCGCTATCTATAAACGC |
| ALID0194 R | GGCAGTACAAAAGCACCC | *lysA* |
| ALID0195 | TCGTCTAACTGGGCACTC |
| ALID0196 | GTGTCTAGTACAGATGTC |
| ALID0197 | CAAGAGGTGCTTCGACCTG |
| ALID0198 | ACATCACCGTCAGGTCTG |
| ALID0199 | TTTCTATCCCTGGATGGC |
| ALID0200 | ATCTTTAGTGAAAACGCC |
| ALID0201 | TGGTGATGAAGAATTTGC |
| ALID0202 F | AATATCAATCAGTATCCG |
| ALID0035 F | ATTCTTTACTTTAGCTTCG | *pyrG* |
| ALID0036 R | CTGACACCTATTAACTCC |
| ALID0037 | CCTGACAGGACTATTCAC |
| ALID0449 F | CCTGACAGTCTAGTACGC | *pyrF* |
| ALID0450 R | TGAATGTTCTGGTAGTCC |
| ALID0451 | ACCAGGACACCAGTCAAG |
